# Identification of a novel sex determining chromosome in cichlid fishes that acts as XY or ZW in different lineages

**DOI:** 10.1007/s10750-021-04560-7

**Published:** 2021-03-13

**Authors:** Anna F. Feller, Vera Ogi, Ole Seehausen, Joana I. Meier

**Affiliations:** 1 Division of Aquatic Ecology & Evolution, Institute of Ecology and Evolution, University of Bern, Baltzerstrasse 6, 3012 Bern, Switzerland; 2 Department of Fish Ecology and Evolution, Centre of Ecology, Evolution and Biogeochemistry (CEEB), Eawag Swiss Federal Institute of Aquatic Science and Technology, Seestrasse 79, 6047 Kastanienbaum, Switzerland; 3 Department of Zoology, University of Cambridge, Downing Street, Cambridge, CB2 3EJ UK; 4 St John’s College, University of Cambridge, St John’s Street, Cambridge, CB2 1TP UK

**Keywords:** Sex determination, Cichlid fishes, Lake Victoria, Lake Malawi

## Abstract

Sex determination systems are highly conserved among most vertebrates with genetic sex determination, but can be variable and evolve rapidly in some. Here, we study sex determination in a clade with exceptionally high sex chromosome turnover rates. We identify the sex determining chromosomes in three interspecific crosses of haplochromine cichlid fishes from Lakes Victoria and Malawi. We find evidence for different sex determiners in each cross. In the Malawi cross and one Victoria cross the same chromosome is sex-linked but while females are the heterogametic sex in the Malawi species, males are the heterogametic sex in the Victoria species. This chromosome has not previously been reported to be sex determining in cichlids, increasing the number of different chromosomes shown to be sex determining in cichlids to 12. All Lake Victoria species of our crosses are less than 15,000 years divergent, and we identified different sex determiners among them. Our study provides further evidence for the diversity and evolutionary flexibility of sex determination in cichlids, factors which might contribute to their rapid adaptive radiations.

**Supplementary Information:**

The online version contains supplementary material available at 10.1007/s10750-021-04560-7.

## Introduction

Most eukaryotes reproduce sexually and individuals are either male producing many small sperm or female producing few large ova (Bell, 1982). One of the most puzzling aspects of sexual reproduction is that while the existence of two sexes is highly conserved, there is a diversity of mechanisms triggering development as either male or female (reviewed in Bachtrog et al., 2014). Among animals with genetic sex determination, genes determining sex have evolved independently many times. Some groups have stable sex chromosomes that remained conserved across millions of years (White, 1977; Ming et al., 2011; Cortez et al., 2014). For example, the mammalian Y chromosome arose approximately 180 million years ago (Cortez et al., 2014), the avian female-determining W chromosome about 140 million years ago (Cortez et al., 2014), and the Lepidopteran W chromosome is more than 180 million years old (Sahara et al., 2012). These ancient sex chromosomes have strongly deteriorated over time and accumulated sexually antagonistic alleles, leading to heteromorphic (morphologically distinct, often degenerated) chromosomes (Bachtrog et al., 2014). In contrast, other taxonomic groups display much faster turnover of sex chromosomes (e.g. frogs Jeffries et al., 2018; fishes Kitano & Peichel, 2012). Only about 10% of fishes have heteromorphic sex chromosomes (Devlin & Nagahama, 2002), and sex determining genes can differ among closely related species or even within a single species (Orzack et al., 1980; Seehausen et al., 1999; Kitano & Peichel 2012; Cheng et al., 2013).

One of the fastest rates of sex determination turnover is found in cichlid fishes (Gammerdinger & Kocher, 2018; Böhne et al., 2019). Many different sex determination systems have been identified involving different chromosomes, including male and female heterogametic systems and polygenic sex determination (Seehausen et al., 1999; Gammerdinger & Kocher 2018; Böhne et al., 2019). In addition, B chromosomes, i.e. accessory chromosomes only found in some individuals of a species, have been suggested to act as female sex determiners in two cichlid species (Yoshida et al., 2011; Clark & Kocher, 2019). The family Cichlidae contains very young species radiations such as those of several hundred species each in Lake Victoria (15,000 years, Bezault et al., 2011; Johnson et al., 2000; McGee et al., 2020) and Lake Malawi (1–5 million years, Genner et al., 2007; Ivory et al., 2016; Malinsky et al., 2018). Even within these young radiations, several different sex determining chromosomes have been identified in the few species whose sex determination systems have been studied to this date. At least five sex determination systems are present in Lake Malawi haplochromine cichlids involving *Oreochromis niloticus* (Linnaeus, 1758) linkage groups (O) 3, 5, 7, and 20 (Gammerdinger & Kocher, 2018; Böhne et al., 2019), and in multiple species, some females have a B chromosome (Clark et al., 2016; Clark & Kocher 2019). Only few species from Lake Victoria have been tested for sex chromosomes. One study identified two QTLs on O2 and O5 in a cross of *Paralabidochromis sauvagei* (Pfeffer, 1896) and *Paralabidochromis chilotes* (Boulenger 1911) (Kudo et al., 2015). A study of a cross between two *Pundamilia* species inferred an XY sex determination system on O23 in a 1.9 Mb region containing the anti-Müllerian hormone gene (*amh*) (Feulner et al., 2018), and a third study discovered the presence of a feminizing B chromosome in *Lithochromis rubripinnis* Seehausen, Lippitsch & Bouton, 1998 (Yoshida et al., 2011).

Theoretical modelling has shown that sex chromosome turnover events can be favoured by deleterious mutation load on the non-recombining chromosome, if the load outweighs the benefits gained by factors favouring maintenance of an initial sex chromosome, including carrying sexually antagonistic genes where males benefit from having one allele, and females another, maintained in linkage disequilibrium (Blaser et al., 2013, 2014). Alternatively, a new sex-determiner can spread if it is physically linked to sexually antagonistic autosomal mutations (van Doorn & Kirkpatrick, 2007; van Doorn & Kirkpatrick, 2010). The invasion of new sex determiners in response to sexually antagonistic selection has been suggested to have occurred in cichlids (Roberts et al., 2009), and this might contribute to rapid and repeated speciation in cichlids (Lande et al., 2001; Kocher, 2004). Finally, novel sex chromosomes can invade if meiotic drive or endoparasites have produced an unequal sex ratio (Kozielska et al., 2010).

**Fig. 1:**
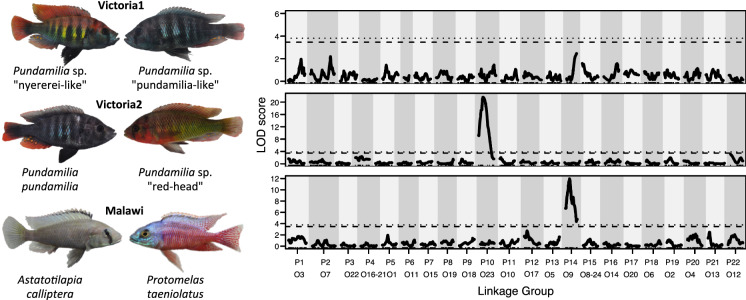
QTL mapping shows different sex determination in each cichlid cross. On the left, representative male individuals of the six parental species used in the three crosses (parental species used as grandmother in the crossing scheme on the left, parental species used as grandfather on the right). On the right, LOD scores of QTL mapping for each cross separately. The linkage groups (shown with alternating grey background shading) are numbered according to the *Pundamilia nyererei* v2 reference (P, Feulner et al., 2018) and the corresponding *Oreochromis niloticus* reference numbers (O, Brawand et al., 2014) below. The results are from standard interval mapping including all families in a cross (without covariates). The dashed lines represent a genome-wide significance threshold of *P* = 0.1, the dotted lines *P* = 0.05. QTL mapping reveals a clear sex QTL on P10/O23 for the Victoria2 cross and on P14/O9 for the Malawi cross, but does not identify a sex QTL for the Victoria 1 cross (see also Table S1)

Here, we identify the sex determination systems in three crosses of closely related species of a clade with exceptionally fast sex chromosome turnover: haplochromine cichlids. We compare two interspecific crosses from Lake Victoria and one interspecific cross from Lake Malawi (Fig. 1). Using QTL mapping and the identification of sex differences based on genotype frequencies we aimed to (1) identify the sex determining chromosomes in the three crosses, (2) test if the sex chromosomes show signs of degeneration, (3) test whether they represent male-heterogametic (XY) or female-heterogametic (ZW) systems and (4) trace back the sex determining alleles to the parental species. Our results reveal multiple sex determiners in our crosses, including a new chromosome involved in sex determination that acts as XY or ZW in different species. Overall, our study adds to a growing body of evidence for high flexibility in sex determination in cichlid fishes.

## Materials and methods

### Experimental crosses

We analysed data from three interspecific second generation (F2) hybrid crosses to infer the sex determination system in each of them. One cross is between the sympatric sister species *Pundamilia* sp. “nyererei-like” and *Pundamilia* sp. “pundamilia-like” (‘Victoria1’; see Feller et al., 2020a), one between the non-sympatric species *Pundamilia pundamilia* Seehausen & Bouton, 1998 and *Pundamilia* sp. “red-head” (‘Victoria2’; see Feulner et al., 2018; Feller et al., 2020a), and one between distantly related *Astatotilapia calliptera* “Chizumulu” (Günther, 1894; population from Chizumulu island, Konings, 2001) and *Protomelas taeniolatus* (Trewavas, 1935) (‘Malawi’; see Stelkens et al., 2009; Selz et al., 2014; Feller et al., 2020b) (Fig. 1).

In both Victoria crosses, the F2 individuals used in our analyses belong to two F1 families (henceforth families A and B). In the Malawi cross the F2 individuals belong to six F1 families, but for most individuals the information to which family they belong was not available. All F2s were reared to an age of at least 1 year in our aquarium system before they were sexed based on colouration and overall morphological appearance, sacrificed (using MS222; 25–50 mg/l for sedation; 300–400 mg/l for euthanization) and fin-clipped. In individuals that showed an inconsistent genetic pattern with phenotypic sex, we additionally inspected the gonads. Furthermore, we performed gonad inspection in ten individuals that were difficult to sex in each cross, which confirmed that sexing based on external phenotype is reliable. All fish were maintained and bred in a large recirculation facility either at the University of Bern (Victoria1 and Victoria2) or at Eawag (Malawi), with a water temperature of 24–26°C and a 12:12 h light/dark cycle.

We tested if the sex ratio found among all surviving F2 offspring (including those not sequenced) deviates from the expected 0.5 ratio by applying a binomial test with the R function ‘binom.test’.

### RAD tag sequencing

As described in Feller et al. (2020a) for Victoria1, in Feulner et al. (2018) for Victoria2 and in Feller et al. (2020b) for Malawi, DNA was extracted from fin-clips (stored in 98% ethanol) using phenol–chloroform (Sambrook & Russell, 2001). Restriction-site associated DNA (RAD) sequencing libraries were prepared following Baird et al. (2008) with some modifications. Prior to enzyme digestion with *Sbf*I, DNA concentrations were normalised for all samples in one library. 4–48 individuals carrying custom 5–8 bp barcodes were pooled into one library. This was followed by shearing (on a Covaris M220 focused-ultrasonicator) and size selection of 300–700 bp fragments (on a SageELF machine). Each library was amplified in four 50 ml aliquots reactions, and the size-selection step was repeated with the SageELF machine or magnetic beads. Single end sequencing (100–125 bp) was done on an Illumina HiSeq 2500 platform at the Genomic Technologies Facility of the University of Lausanne or at the Next Generation Sequencing Platform of the University of Bern, using one lane per library. To increase complexity in the first 10 sequenced base pairs, and for base quality recalibration (see below), 4–12.5% PhiX genomic DNA was sequenced together with each library.

### Sequence processing

As described in Feller et al. (2020a) for Victoria1 and Victoria2 and in Feller et al. (2020b) for Malawi, PhiX reads were removed with Bowtie2 v2.3.2 (Langmead & Salzberg 2012). Reads were demultiplexed and trimmed to 85–90 bp with process_radtags implemented in stacks v.1.40 (Catchen et al., 2013). Single errors in the barcode were corrected and reads with incomplete restriction sites discarded. This was followed by filtering reads for a minimum quality of 10 at all bases and of 30 in at least 95% of the reads using the FASTQ quality filter (http://hannonlab.cshl.edu/fastx_toolkit/index.html). Bowtie2 v2.3.2 (Langmead & Salzberg 2012) was used for alignment to the anchored version of the reference genome of a male *Pundamilia nyererei* (Witte-Maas & Witte, 1985) (Feulner et al., 2018), allowing one mismatch. Base quality recalibration based on the PhiX reads was done per library using the GATK BaseRecalibrator and PrintReads modules (McKenna et al., 2010). For genotyping, GATK Unified Genotyper v3.7 (McKenna et al., 2010) was used (minimum base quality score set to 20). In Victora1 and Victoria2 only uniquely aligned reads were used.

The resulting vcf files were filtered using bcftools implemented in samtools v.1.8 and v1.9 (Li et al., 2009) and using vcftools v.0.1.14 and v.0.1.16 (Danecek et al., 2011) as described in Feller et al. (2020a) for both Victoria crosses and also applied to the Malawi cross: Sites with > 50% missing data and individuals with a mean depth of < 12 or > 50% missing data were excluded. Only bi-allelic SNPs with a mean sequencing depth of less than 1.5 times the interquartile range from the mean were kept and sites within 10 bp of indels were removed. Genotypes with a depth of < 10 were set to missing. Individuals were excluded if the heterozygous read balance was heavily skewed (indicating PCR duplicates). Sites were then again filtered for no more than 50% missing data and for a minor allele frequency of at least 0.05.

This resulted in a dataset with 10,598 SNPs and 224 individuals (218 F2s, 4 F1, 2 F0) for Victoria1, in 9990 SNPs and 192 individuals (186 F2s, 4 F1, 2 F0) for Victoria2, and in 12,187 SNPs and 126 individuals (114 F2s, 12 parental species individuals) for Malawi.

### Linkage map construction

As described in Feller et al. (2020a) for both Victoria crosses and also applied to the Malawi cross, linkage maps for all three crosses were constructed in JoinMap 4.0 (Van Ooijen, 2006) after applying an allelic balance correction (https://github.com/joanam/scripts/allelicBalance.py) to the grandparents (F0) and then subsetting the datasets to SNPs that are fixed for alternative alleles (homozygous alternative) between the F0. In Victoria1, 954 SNPs were homozygous in the F0 grandmother and heterozygous in all 4 parents (F1s) (no missing data allowed). This set differs somewhat from the published map in Feller et al. (2020a) because further analyses (see below) revealed inconsistencies in the F0 grandfather’s genotype, and we thus only used the F0 grandmother and the F1s to filter for putative homozygous alternative SNPs, and we applied a more stringent segregation distortion filter (*P* < 0.01) during linkage map construction. In Victoria2, 2358 SNPs were homozygous alternative in the F0 and heterozygous in 2 F1s (no missing data allowed; the other 2 F1s were removed in the filtering process). In the Malawi cross, fin-clips and sequences for the F0 and F1 of this cross were not available. Instead, as described in Feller et al. (2020b), 5–6 individuals of each parental species were sequenced (*A. calliptera* “Chizumulu”: 3 males, 2 females, 1 undeterminable (all from our lab population); *P. taeniolatus*: 4 males (two from our lab population and two from two aquarium fish breeders), 1 female (from one of the aquarium fish breeders)). 1775 SNPs were retained as homozygous alternative between the two parental species.

In linkage map construction, SNPs with extreme segregation distortion (*P* < 0.001) or with > 20% missing genotypes, were excluded. Identical SNPs were removed (i.e. SNPs within the same RAD locus; > 0.950). Individuals with > 30% missing data were excluded. The Victoria1 linkage map was generated from 216 F2 individuals (173 males, 43 females), the Victoria2 linkage map from 171 F2 individuals (115 males, 56 females), the Malawi linkage map from 108 F2 individuals (34 males, 66 females, 8 juveniles with undetermined sex). Linkage groups were identified based on a LOD threshold of 5–6, excluding loci with a recombination frequency of above 0.6. The strongest cross-link (SCL) values in the maps are 4.6–5.8. The Kosambi regression mapping algorithm was used to build the linkage maps (LOD threshold 1.0, recombination threshold 0.499, goodness-of-fit threshold 5.0, no fixed order). Two rounds of mapping were performed with a ripple after addition of each marker to the map.

### QTL mapping

QTL mapping was performed in R/qtl (Broman et al., 2003). The calc.genoprob function was used to calculate conditional genotypes with a fixed step-size of 1 cM, an assumed genotyping error rate of 0.05, and the Kosambi map function. We performed standard interval mapping with the binary model, and determined significance thresholds by (*n* = 1000) permutations. Bayesian credible intervals (95%) were calculated with the ‘bayesint’ function and percentage of variance explained (PVE) was calculated as 1 – 10^−2*LOD/n^ (Broman & Sen, 2009), where LOD is the highest LOD score, and n the number of individuals.

In both Victoria crosses, we additionally performed the analysis with family as covariate, and for each family separately. Where a significant QTL for sex was found, we repeated the mapping excluding the linkage group containing the QTL to test for additional QTLs.

The number of F2 individuals used in QTL mapping (i.e. with phenotypic and genotypic data) are the following: 217 in Victoria1 (172 males (*138 family A *+* 34 family B*) and 45 females (*16 family A *+* 29 family B*)), 186 in Victoria2 (130 males (*76 family A *+* 45 family B *+* 9 family unknown*) and 56 females (*47 family A *+* 9 family B*)), 105 in Malawi (36 males and 69 females).

For markers not mapped to chromosomes, the QTL mapping approach provides only cM positions and LOD scores. For comparison with other statistics, we thus had to infer the bp positions for these markers. We fitted a cubic smoothing spline on cM and bp positions for markers mapped to chromosomes with the smooth.spline R function (stats R package v. 3.6.1) with the smoothing parameter (spar) set to 0.8 and predicted bp positions for markers not mapped to chromosomes with the R function predict.smooth.spline.

### Assessing sequencing depth differences between F2 males and F2 females

If the sex chromosomes were heteromorphic due to degeneration of the Y or W chromosome, we would expect differences in sequencing depth between males and females at the sex chromosome. That is, parts of the Y or W chromosome that diverged strongly may no longer map to the corresponding chromosome in the reference genome and some parts may be missing due to deletions in the Y or W chromosome, and this should result in lower sequencing depth in the heterogametic sex (males if XY or females if WZ system). To test this we computed mean sequencing depth in F2 males and females separately with vcftools v 0.1.15 (Danecek et al., 2011). As sequencing depth is quite variable in general, we visualized sex differences in sequencing depth by averaging across 5 Mb windows with the function ‘winScan’ in the R package windowscanr (https://github.com/tavareshugo/WindowScanR).

### Identifying sex chromosomes with differences in genotypes frequencies between F2 males and F2 females

**Fig. 2:**
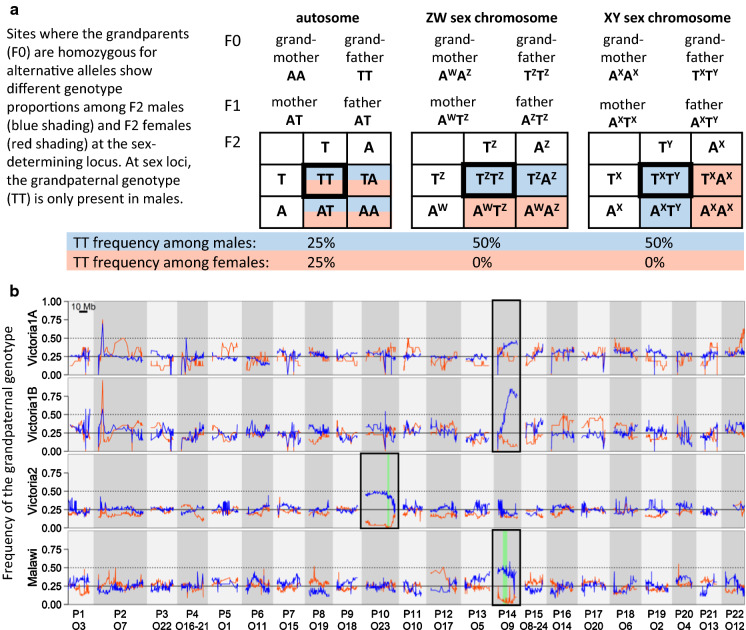
Genotype frequencies support the sex chromosomes identified with QTL mapping and reveal a sex determiner for Victoria1. **a** Punnet squares showing expected genotype frequencies of F2 males (blue background) and F2 females (red background) at sites where the F0 grandparents are homozygous for alternative alleles. For autosomes, the expected frequency of either homozygous genotype is 25% for both sexes. For sex chromosomes, the grandpaternal genotype (TT) should have a frequency of 50% for male offspring and 0% for female offspring. **b** Frequencies of the grandpaternal genotype among F2 males (blue) and females (red) at sites where the F0 grandparents are homozygous for alternative alleles. Different linkage groups are shown with alternating grey background shading and are numbered according to the *Pundamilia nyererei* reference (P) and the *Oreochromis niloticus* reference (O) below. Chromosomes consistent with sex-determination are framed in black. Green vertical bars indicate the location of the 95% Bayesian confidence intervals obtained in QTL mapping analyses. For Victoria1, the two families are shown separately as they differ in their sex determination patterns. Note that among males of Victoria1B, the grandfather genotype exceeds the expected 50% frequency. This is because heterozygotes with one Z from the grandmother and one Z from the grandfather are strongly underrepresented (see Fig. S3)

Next, we computed genotype frequencies to identify the sex determining chromosomes. In order to reduce missing data and correct potential genotyping errors, we phased the dataset with Beagle v. 5.1 (Browning & Browning, 2011). Given that we did not find sex differences in sequencing depth, we assume that reads of both sex chromosomes (X + Y or W + Z) align to the reference genome. We can thus use genotype frequencies to infer sex linkage. Patterns of genotype frequencies are expected to differ between the sexes at the sex determining region. At sites where both F1 parents are heterozygous, both homozygous genotypes should be present at a frequency of 25% in both sexes on autosomes, whereas at sex determining chromosomes, the grandpaternal homozygous genotype should only be found in males and the grandmaternal one only in females (Fig. 2a). Therefore, comparing the proportion of a specific homozygous genotype in male and female offspring should be informative for identifying sex chromosomes. For each cross, we computed the genotype frequencies for the F0 (grandparents), F1 (parents) and the F2 (offspring) separately using vcftools with the option *hardy*. We then extracted bi-allelic sites where the F1 were heterozygous and computed the frequency of the grandparental homozygote genotype among F2 males and females (see Fig. 2a). For the Victoria1 cross, we extracted sites where all four F1s were heterozygous. As we did not have the genotypes of the F1s for the Malawi cross and only low quality F1 genotypes in the Victoria2 cross, we determined sites that are likely heterozygous in both parents by filtering for an allele frequency of 0.45 to 0.55 averaged across both sexes. To avoid including paralogous regions, i.e. reads from duplicated genomic regions that map to a single region on the reference genome, in each cross we excluded all sites with heterozygosity above 75% in the F2 offspring. Because the families of Victoria1 differed both in sex ratio and in the presence of a QTL, we computed the genotypes frequencies of F2 males and females for each family separately.

### Single marker regressions

In the Victoria1 cross, we only found evidence for a sex determining region in the genotype frequency analysis (and this was much weaker in family A than in family B), but not in the QTL analyses. One reason for this could be that a large part of P14 (i.e. the second half) is missing in the linkage map due to segregation distortion. Hence, we performed additional single marker regression analyses to screen for associations of markers not included in the linkage map with sex in the two families. For this, we additionally filtered the 10,598 SNPs for HWE proportions with *P* > 0.1 (only within F2s) using bcftools (Li et al., 2009), resulting in 4695 SNPs over the 22 chromosomes. For each family separately, we then performed an ANOVA for each marker in R, and from this calculated LOD scores as (*n*/2)*(log10(*F*(df/(*n* − df − 1)) + 1), where n = number of individuals and df = degrees of freedom (Broman & Sen, 2009) and PVE (see above). This included 4090 markers (of which 590 on scaffolds) in family A (138 males and 16 females), and 4400 markers (of which 620 on scaffolds) in family B (34 males and 29 females). *P*-values of the ANOVAs were corrected for multiple testing using the Bonferroni method.

The single marker regressions were also run for Victoria2 and Malawi to show that the method works and to check for the presence of additional sex determining regions. In Victoria2, this included 5506 markers (of which 647 on unmapped scaffolds) in family A (74 males, 49 females) and 5531 markers (of which 648 on unmapped scaffolds) in family B (46 males, 8 females). In Malawi this included 9311 markers (of which 1567 on unmapped scaffolds) (35 males, 70 females, no family information available).

### Assessing heterozygote frequency differences between males and females to infer the heterogametic sex

Next, we wanted to infer whether a putative sex chromosome is male or female heterogametic, i.e. XY or ZW. We would expect that the Y is more divergent from the X than the X chromosomes are from each other and likewise, the W should be more divergent from the Z than the Z chromosomes are from each other. Given that there is no difference in sequencing depth between the sexes on any chromosome, the heterogametic sex is expected to show a higher heterozygote frequency (i.e. proportion of individuals that are heterozygous) at the sex chromosome than the homogametic sex. This is because any difference between X and Y should increase heterozygote frequency in males compared to females which have two more similar X chromosomes. Similarly, any difference between Z and W should increase heterozygote frequency in females Therefore, higher heterozygote frequency in males compared to females should indicate an XY system, whereas higher heterozygote frequency in females should indicate a ZW system. We computed heterozygote frequency for male and female offspring separately using the genotype frequencies computed above.

To identify significant sex differences in heterozygosity, we performed 10,000 permutations of the sex assignments and reran the heterozygote frequency calculations for each permutated dataset at all sites. This approach accounts for the exact number of samples available for each sex and also accounts for differences in the total number of heterozygotes among sites. We considered a locus to show a significant sex difference in heterozygote frequency if at most one of the 10,000 permutations were as extreme or more extreme than the observed value (one-sided empirical *P* value < 0.0001).

### Tracing sex determining alleles back to the parental species

In order to identify which species in a cross contributed the sex determining allele(s) in genomic regions identified by the methods outlined above, we inspected genotypes in the F2 individuals at sites that are heterozygous in the heterogametic sex (i.e. sites with Y- or W-linked alleles). We extracted bi-allelic sites where the F0 and F1 individuals of the putative heterogametic sex were heterozygous and the F0 and F1 individuals of the putative homogametic sex were homozygous. F2 individuals of the heterogametic sex are expected to be heterozygous at these sites, thus indicating the presence of the W or Y-linked alleles in heterozygous F2 individuals. For the Malawi cross, we had no sequence data of the grandparents and parents, but RAD data of conspecific individuals of the grandparents. Therefore, we extracted sites where the male individuals (the putatively heterogametic sex) of the species used as grandfather, *P. taeniolatus,* were heterozygous and those of the species used as grandmother, *A. calliptera*, were homozygous. For all crosses, we visualized the genotypes for each F2 individual at these sex-linked sites in yellow for homozygotes of the allele derived from the grandfather (Z or Y), orange for heterozygotes (WZ or XY) and red for homozygotes for the allele derived from the grandmother (W or X). To aid visualization, genotypes likely representing genotype errors (e.g. caused by allelic dropout) were replaced by the adjacent genotypes. For this, runs of up to three equal genotypes that differed from the five genotypes on both sides were replaced by the adjacent genotypes.

This analysis revealed that in both families of the Victoria1 cross some males had a W allele and some females did not have a W allele, and in the Malawi cross some females had a Y allele. Therefore, we assessed if the parental lineage origin of the Z or X alleles, respectively, played an additional role in sex determination. For this analysis, we extracted the most strongly sex-linked site with homozygous F0 and heterozygous F1 individuals and the most strongly sex-linked site with heterozygous parents and grandparents of the heterogametic sex. The combination of the genotypes at these two sites allowed us to trace back each of the alleles to the grandparents and to determine the combination of X/Y or Z/W alleles of each individual.

Knowing the combination of sex chromosomes of each individual, we then computed the divergence between the different sex chromosomes as heterozygosity, i.e. the proportion of sites that differ between the two chromosomes among all positions sequenced, for individuals with different combinations of sex chromosomes. For this, we generated a vcf file with all individuals of all three crosses together, calling both monomorphic and polymorphic sites. We removed sites with more than 25% missing data, genotypes with less than 10 reads and SNPs with only one or two allele copy counts (likely sequencing errors). Individuals with more than 25% missing data proportion or recombinants at the sex determination region were removed. We used the Python script by Simon Martin (https://github.com/simonhmartin/genomics_general/popgenWindows.py) to compute heterozygosity for each individual and divergence (*d*_xy_) between the Victoria 1 and Victoria 2 cross individuals in windows of 1 Mbp.

## Results

### Sex ratios among F2 offspring

Of all the Victoria1 (sympatric sister species) F2 offspring that survived to adulthood 37% were females in family A (85 females, 144 males), and 78% were females in family B (114 females, 32 males). This sex ratio strongly differs from the expected 50% with binomial test *P*-values of < 0.001 for both families. In Victoria2 (non-sympatric species), both families had even sex ratios (91 males and 100 females in family A, 48 males and 34 females in family B; binomial test *P* > 0.15 in both families). There is a female-bias in the Malawi cross (51 males, 102 females; binomial test *P* < 0.001).

### QTL mapping results

We found no significant QTLs for sex in the Victoria1 cross, neither in the analyses including both families with or without accounting for an effect of family by adding it as covariate in the analyses, nor in the analyses for both families separately (Fig. 1).

The presence of a significant QTL for sex on linkage group P10/O23 in the Victoria2 cross previously reported by Feulner et al. (2018) is also supported by our analyses with a slightly modified linkage map and with a different number of individuals (Fig. 1, Table S1; *P* < 0.001, LOD = 21.58, PVE = 41.39), and also when addtionally accounting for an effect of family by adding it as covariate in the analyses (Table S1). The results were also very similar when the mapping was performed for each family separately (Table S1). The location of the QTL is slightly shifted and the association is less strong in the second family, but considering the small number of individuals, these differences may be due to sampling variance. Repeating the QTL mapping excluding P10/O23 revealed no further QTLs.

In the Malawi cross we found a significant QTL for sex on P14/O9 (Fig. 1, Table S1; *P* < 0.001, LOD = 11.96, PVE = 40.84). The Bayesian confidence interval of 7.27 cM covers three markers and spans a region of 5.76 Gb. Pedigree information was not available for this cross. Repeating the QTL mapping excluding P14/O9 revealed no further QTLs.

### Sequencing depth differences between males and females

We did not find strong differences in sequencing depth on any chromosomes in any cross (Fig. S1). The ratio of sequencing depth of females divided by males of all 5 Mb window averages range from 0.93–1.10, 0.92–1.09, 0.90–1.08, and 0.92–1.12 in the Victoria1A, Victoria 1B, Victoria2 and Malawi cross, respectively. The 5 Mb window averages at putative sex chromosomes are also very close to 1 (Victoria1A: 0.998–1.086, Victoria1B: 0.959–1.091, Victoria2: 0.954–1.047, Malawi: 0.953–1.046). This indicates that X and Y reads or W and Z reads map to the reference genome, and thus that any potential sex determining chromosomes are not (yet) heteromorphic due to degeneration.

### Difference in genotype frequencies between F2 males and F2 females support putative sex chromosomes

In each cross, the frequency of the grandpaternal homozygous genotype shows clear frequency differences between the sexes on a single chromosome, indicating that this chromosome carries a sex determiner (Fig. 2b). The chromosomes P10/O23 and P14/O9 which are identified as sex determining in the Victoria2 and Malawi cross, respectively, are supported by grandpaternal genotype frequencies of close to 50% in males and 0% in females. In the Victoria1 cross, the two families differ in the genotype frequencies. Both families show sex differences in genotype proportions on chromosome P14/O9 but the difference is much stronger in family B. In family B, 28 of the 33 males (85%) have the grandpaternal genotype exceeding the expected 50% frequency (Fig. S3).

### Additional single marker regressions confirm the observed patterns

In single-marker regressions performed for each Victoria1 family independently, no marker was associated with sex in family A after correcting for multiple testing (Fig. S2). In family B, several markers on P14/O9 were significantly associated with sex after correcting for multiple testing. The markers that are associated with sex on P14/O9 are located at the end of P14 (between chr14_12466922 - chr14_25950108, which is indeed the region missing markers in the linkage map). The same analyses in Victoria2 and Malawi show the same clear peaks already seen in the standard QTL mapping analyses (compare Fig. 1 with Fig. S2). The two families of Victoria2 show similar sex-associations. The few additional significant markers on P22/O12 in Malawi may be artefacts that could be due to the fact that several families were combined, since we have no other evidence of sex linkage of this chromosome in this cross.

### Differences in heterozygote frequencies between the sexes support the putative sex chromosomes and reveal which sex is heterogamous in each cross

**Fig. 3:**
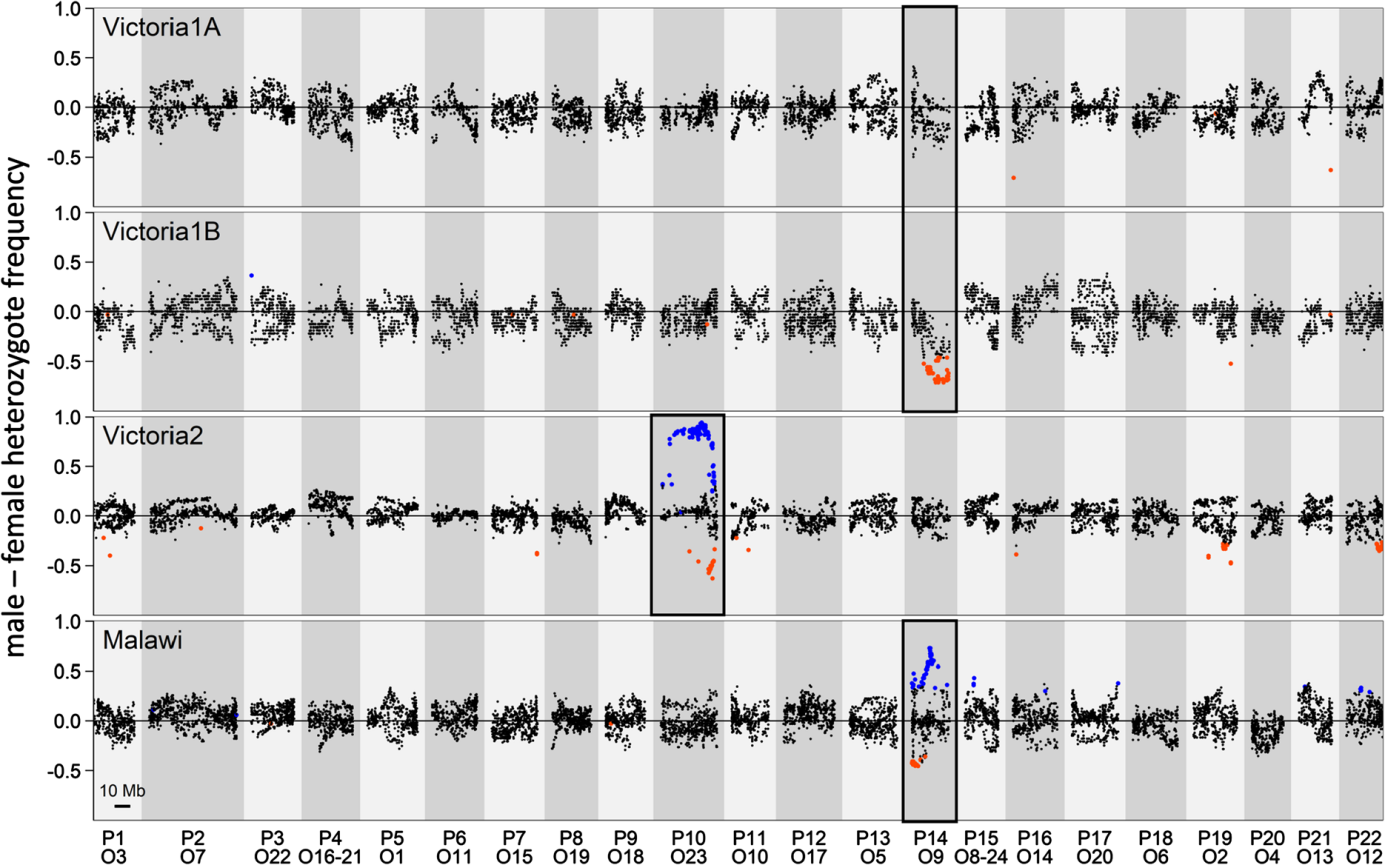
Sex differences in heterozygote frequency support the previously identified sex determining regions and reveal male and female heterogamety. Proportion of heterozygotes among male minus the proportion of heterozygotes among female F2 individuals shows a clear deviation from 0 at putative sex chromosomes (framed in black). Deviations from 0 greater than at least 1/10,000 permutations (empirical *P*-value < 0.0001) are shown with larger symbols. Significantly negative heterozygote frequency differences are highlighted in red and indicate that more females than males are heterozygous at these sites. Significantly positive heterozygote frequency differences are highlighted in blue. Chromosomes inferred to be sex-determining in previous analyses are highlighted with a black frame. The heterozygosity frequency differences suggest female heterogamety in the Victoria1 cross and male heterogamety in the Victoria2 and in the Malawi cross

**Fig. 4:**
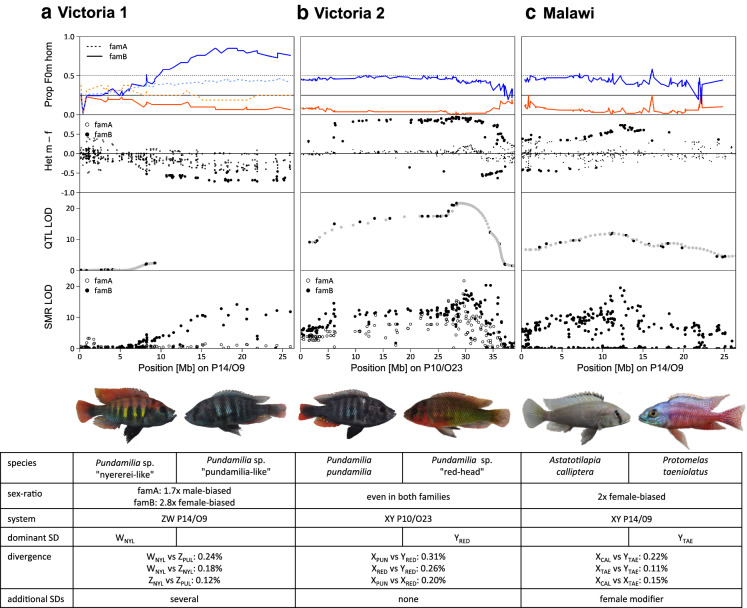
Putative sex chromosomes are supported by multiple lines of evidence. Three lines of evidence support the location of a sex determiner on the putative sex chromosomes in the three crosses: top: Proportion of individuals that are homozygous for the grandfather-derived allele (F0m hom) are shown for females (red) and males (blue). Second row: Sex difference in the proportion of heterozygotes indicates female heterogamety (ZW) in Victoria1 and male heterogamety (XY) in the two other crosses. Sex differences in heterozygote proportions with empirical *P*-value < 0.0001 are highlighted with larger points. Third row: LOD scores from QTL mapping analyses. Markers on unmapped scaffolds and interpolated markers (where genotypes are inferred as implemented in the calc.genoprob function in R/qtl (Broman & Sen, 2009)) are shown in grey with bp positions predicted from local recombination rates. Note that the Victoria1 QTL LOD scores could not be computed for a large part of the chromosome as the markers in the second half were filtered out due to segregation distortion. This is likely due to the strongly skewed sex ratios in this cross. Bottom: LOD scores of single marker regressions (smr) performed for each family separately. The table below gives an overview of the sex determining systems and sex determiners (SD) found in each cross, and species, respectively. For the divergence estimates between the sex chromosomes see also Fig. S6

The chromosomes previously identified as carrying a sex determiner showed many sites with sex differences in heterozygote frequency (Fig. 3). Family B of the Victoria1 cross showed a higher heterozygote frequency in females than in males, whereas this effect was weaker and not significant in family A. The Victoria2 and Malawi crosses showed a higher heterozygote frequency in males than in females (Fig. 3). We thus conclude that the Victoria1 cross is female heterogametic (ZW) and the Victoria2 and Malawi crosses are male heterogametic (XY) (Figs. 3, 4).

### Tracing back the dominant sex determiners to the parental species

**Fig. 5:**
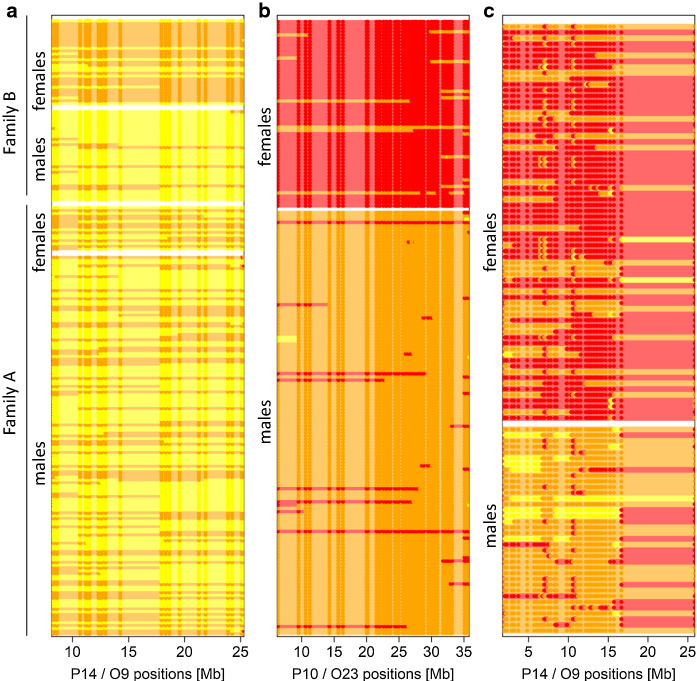
Genotypes at sites with Y or W-linked alleles. Genotypes of the F2 individuals are visualized as red for homozygotes of the grandmother-derived allele, orange for heterozygotes and yellow for homozygotes of the grandfather-derived allele. **a** Victoria1 sites where the F0 and F1 females are heterozygous. Heterozygotes (orange) carry the W allele from the *P*. sp. “nyererei-like” grandmother (W_NYL_). Most females of family B have a W allele and most males of family B do not. However, the sex-specific difference is smaller in family A. **b** Victoria2 sites where the male F0 and F1 are heterozygous. Except for three individuals, all males and no females are heterozygous and thus carry the Y-allele from the *P*. sp. “red-head” grandfather (Y_RED_). **c** Malawi sites where the grandfather surrogates are heterozygous. Heterozygotes carry the Y-allele from the *P. taeniolatus* grandfather (Y_TAE_). All males carry one or even two Y alleles, and most females have no Y allele. For more details see Figs. S3–S5

In family B of the Victoria1 cross, 24 of the 31 females (77.4%) carry the W allele derived from *P*. sp. “nyererei-like” (W_NYL_) and only 3 of 33 males (0.09%) carry a W_NYL_ allele (Figs. 5a, S3, S5). Of the 30 ZZ males, 28 (93.3%) carry two Z alleles derived from the *P*. sp. “pundamilia-like” grandfather (Z_PUL_Z_PUL_), and only two males carry one Z allele from the grandfather and one from the grandmother (Z_NYL_Z_PUL_). This difference is not apparent among ZZ females (five Z_NYL_Z_PUL_, two Z_PUL_Z_PUL_). Individuals with Z_NYL_Z_PUL_ are generally underrepresented (12% of all individuals, sex-ratio corrected). It is thus possible that males, but not females, with two different Z alleles (Z_NYL_Z_PUL_) are less viable (Fig. S3).

In family A of the Victoria1 cross, the W allele derived from *P*. sp. “nyererei-like” (W_NYL_) is also more common among females than among males but the sex association is much weaker (Fig. 5a). There is likely more than one additional sex determiner or modifier segregating in this family, but with only 16 females (and 138 males), we lack power to identify them. The lack of individuals with two Z chromosomes from different parental species (Z_NYL_Z_PUL_) is not observed in family A. However, it is possible that family A and family B inherited different Z chromosomes from their *P*. sp. “pundamilia-like” grandfather and that only one of them interacts negatively with Z_NYL_.

In the Victoria2 cross, there are only two of 186 individuals where the phenotypic sex assignment did not match the genotype at these sites (Fig. 5b). We can thus conclude that there is likely only a single sex determiner on chromosome P10/O23 with a dominant Y-allele derived from the *P.* sp. “red-head” grandfather (Y_RED_).

In the Malawi cross, all males carry a Y-allele at chromosome P14/O9 derived from the *P. taeniolatus* grandfather (Y_TAE_; Figs. 5c, S3, S4). Three males even carry two Y-alleles (Y_TAE_Y_TAE_), indicating that their (F1) mother also had a Y-allele. However, 14 of the 68 females (20%) also carry a Y_TAE_-allele. The presence of ovaries in these individuals has been confirmed with gonad inspection. Thirteen of these females combine the *P. taeniolatus*-derived Y_TAE_ with an X_CAL_ allele derived from the *A. calliptera* grandmother (X_CAL_Y_TAE_) and only one has the grandpaternal combination of X_TAE_Y_TAE_, which could indicate a feminzing effect of the X_CAL_ allele (Figs. S3, sS4). However, many males have the same X_CAL_Y_TAE_ allele combination. We must therefore assume the presence of an additional female modifier, likely derived from *A. calliptera*, interacting with X_CAL_. An additional female modifier is also consistent with the finding of a female-biased sex ratio in this cross.

The divergence between the sex chromosomes is greater in heterogametic than in homogametic individuals in all crosses (Figs. 4, S6). However, the differences are small and the divergence between different sex chromosomes from the same parental species (W_NYL_Z_NYL_, X_RED_Y_RED_, X_TAE_Y_TAE_) is similar to the divergence between the Lake Victoria cichlid species of the different crosses which are less than 15,000 years divergent (Fig. S6). Therefore, we conclude that the sex chromosomes have likely diverged very recently.

## Discussion

**Fig. 6:**
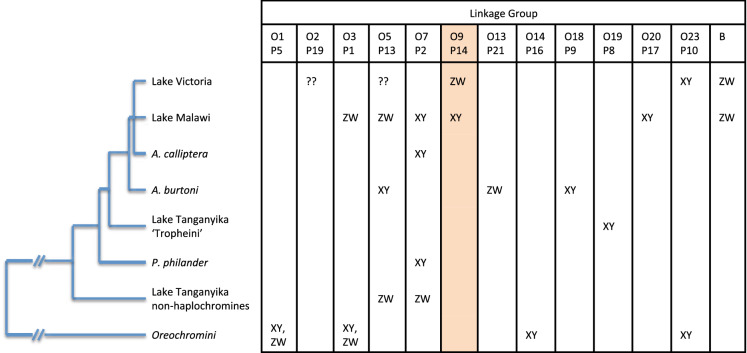
Chromosome P14/O9 has not previously been reported to be sex determining in East African cichlids. The table shows an updated overview of sex determination systems in East African cichlids based on information summarised in Böhne et al. (2019). Linkage group numbers according to the *Oreochromis niloticus* (O) and *Pundamilia nyererei* (P) references. XY, ZW and “??” refers to male, female, or unknown heterogamety, respectively. Phylogenetic relationships are shown schematically to the left of the table redrawn from Meyer et al. (2016). Chromosome P14/O9 (highlighted in orange) is sex-determining in the Victoria1 cross and in the Lake Malawi cross. It has not been found to be sex determining in any other East African cichlid species before

In our analyses of three interspecific crosses of haplochromine cichlids from Lakes Victoria and Malawi we find different sex determining chromosomes and systems even between the species from Lake Victoria which are all less than 15,000 years divergent. This highlights the high variability in sex determination in haplochromine cichlid fishes, consistent with previous findings (Holzberg, 1978; Seehausen et al., 1999; Lande et al., 2001; Gammerdinger & Kocher 2018; Böhne et al., 2019). Furthermore, we report a chromosome to be involved in sex determination that has not previously been reported as such in East African cichlids (Fig. 6). This chromosome contains a sex determiner in one of the Lake Victoria species and in one of the Lake Malawi species that are ~ 2.5 My divergent, though possibly on different regions of the chromosome and with a different heterogametic sex (Fig. 4).

### Using interspecific crosses to infer sex determination

Here, we identified sex determiners in interspecific crosses with one set of grandparents. This allowed us to identify dominant sex determiners contributed by one of the grandparents. In the sympatric Victoria cross (Victoria1), we identified a dominant female determiner from the *P*. sp. “nyererei-like” grandmother (W_NYL_) on chromosome P14/O9 (Fig. 4). Therefore, we can conclude that in *P*. sp. “nyererei-like” sex is determined by a ZW system on P14/09, but we cannot test if it is fixed in *P*. sp. “nyererei-like” or if this sex determiner also exists in the second grandparental species, *P*. sp. “pundamilia-like”. In the non-sympatric Victoria cross (Victoria2), we identified a dominant male determiner from the *P*. sp. “red-head” grandfather (Y_RED_) on chromosome P10/O23. We did not find any evidence for a dominant female sex determiner. Therefore, it is likely that in *P. pundamilia*, the grandmaternal species, sex is also determined by an XY system, but it is unclear if the same chromosome (P10/O23) is sex determining. In the Malawi cross, a dominant male determiner from the *P. taeniolatus* grandfather (Y_TAE_) is located on chromosome P14/O9. We found evidence for an additional female determiner in this cross which could have been contributed by the grandmaternal species *A. calliptera*. While interspecific crosses allow the detection of dominant sex determiners that can be traced back to one of the grandparental species, additional intra- or interspecific crosses with different sets of grandparents and reciprocal crossings would be needed to infer whether these sex determiners exist in both crossed species.

### Multiple sex determiners

In two of our interspecies crosses, the Malawi and the Victoria1 cross, our results suggest the presence of multiple sex determining loci or sex modifiers. However, as the crossed species could have different sex determiners, this does not necessarily mean that multiple sex determiners exist in a single species. A sex determination locus in *A. calliptera*, the species used as grandmother for our Malawi cross, has already been mapped to P2/O7 and identified as a male heterogametic XY system (Peterson et al., 2017). Our *A. calliptera* grandmother should thus have been XX on P2/O7 and should not have contributed a dominant sex determination allele. Consistent with this, we found a different sex determining region on P14/09 for which the Y-allele can be traced back to the *P. taeniolatus* grandfather (Y_TAE_). However, a simple XY-system cannot explain the female-biased sex ratio in this cross and the fact that 20% of the females carry a Y chromosome (most of which are X_CAL_Y_TAE_). It is thus likely that a female determiner or modifier interacting with X_CAL_ exists. Alternatively, Y_TAE_ may show incomplete penetrance in the presence of X_CAL_, comparable to the findings of (Parnell & Streelman, 2013), who found a complex polygenic system that determined sex in a hierarchical fashion in their interspecific cross.

Multiple sex determining loci likely also segregate in our Victoria1 cross of very closely related sympatric species. In this cross, the W-allele in the identified sex determining region on P14/O9 can be traced back to the *P*. sp. “nyererei-like” grandmother (W_NYL_). However, the two families differ in the strength of this correlation of W_NYL_-presence with sex, and also feature highly distorted sex ratios (in opposite directions). Both of these are indications for the presence of additional sex determiners. In the female-biased family B, males that combine Z chromosomes from the different grandparental species (Z_NYL_Z_PUL_) are strongly underrepresented which could indicate reduced survival of these males (see also below). Additional sex determination loci must be present in both families (see Fig. S3). The two species of this cross diverged only a few hundred years ago in sympatry (Meier et al., 2017). Given their recent speciation and continued presence of gene flow during speciation it may seem unlikely that they could have evolved different sex determiners. However, these species are derived from a hybrid population between two older species (*P. nyererei* and *P. pundamilia* (Meier et al., 2017)), both of which may have contributed distinct sex determiners and/or sex modifiers. One of the parental species of the hybrid population from which the two younger species evolved, *P. pundamilia*, was used as the grandmother in our Victoria2 cross. In this cross, we only found a single sex determination locus with a male determiner on P10/O23 that came from the grandfather (Y_RED_, *P*. sp. “red-head”). It is thus unlikely that our *P. pundamilia* grandmother shared the dominant female determiner on chromosome P14/O9 with *P*. sp. “nyererei-like”. Our findings are consistent with different sex determiners in *P. nyererei* and *P. pundamilia* which gave rise to the hybrid swarm from which *P*. sp. “nyererei-like” and *P*. sp. “pundamilia-like” evolved. A next step would be to sequence population samples to test if the sex determiners represent fixed species differences or if multiple sex determiners segregate in some of the species.

While polygenic sex determination can be an evolutionary stable strategy in some cases (Moore & Roberts, 2013), it is more commonly thought to be a transient state between an ancestral and derived sex determination system (Rice, 1986; Van Doorn, 2014). This is because selection against sex ratio distortion or a fitness advantage of one of the sex determiners is expected to lead to the fixation of a single sex determiner (Orzack et al., 1980; Bull, 1983; Lande et al., 2001; Moore & Roberts 2013; Van Doorn, 2014). Nevertheless, the presence of multiple sex determiners has been demonstrated in the more distantly related haplochromine cichlid *Astatotilapia burtoni* (Günther, 1894), where at least three different chromosomes carry sex determining loci (Böhne et al., 2016; Roberts et al., 2016). Multiple sex-determining loci are likely also segregating in some Lake Malawi cichlid species (e.g. Parnell & Streelman, 2013). If sex determination is indeed commonly polygenic in East African cichlids, multiple crosses with different sets of grandparents are required to identify the different sex determiners. Moreover, larger numbers of individuals would be needed to detect additional weaker sex determining loci. A second sex determiner could be located on a B chromosome and would thus be even harder to detect. The presence of feminizing B chromosomes has been confirmed in other cichlids (Yoshida et al., 2011; Clark & Kocher, 2019). In family B of the Victoria1 cross, the females may either carry a dominant female determiner on linkage group P14/O9 or a B chromosome, explaining the female-biased sex ratio. Similarly, a feminizing B chromosome could explain the female-biased sex ratio in the Malawi cross and the presence of females with a Y chromosome. Whole-genome sequencing or the detection of B-chromosomal markers (as in Clark et al., 2018) would be needed to test this hypothesis.

### Same chromosome: different heterogametic sex

In two crosses we have identified the same chromosome (P14/O9) as sex determining but with an XY system in *Protomelas taeniolatus* from Lake Malawi, and a ZW system in *Pundamilia* sp. “nyererei-like” from Lake Victoria. Similar findings have been made in other cichlids, where the same chromosome acts as XY or ZW system in different lineages. Chromosome P13/O5 is an XY sex chromosome in *A. burtoni* (Böhne et al., 2016; Roberts et al., 2016) and ZW in several Lake Malawi species (Ser et al., 2010), and similarly, P2/O7 is an XY sex chromosome in *A. calliptera* and other species from Lake Malawi (Ser et al., 2010; Peterson et al., 2017) and ZW in *Hemibates stenosoma* (Boulenger, 1901) from Lake Tanganyika (Gammerdinger et al., 2018). Theoretical studies suggest that transitions from an XY to a ZW system or vice versa could happen readily in the presence of sex ratio distortion, which in turn can be caused by meiotic drive elements (Scott et al., 2018) or by a new sex determiner that can spread if it is under sexual selection (Lande et al., 2001; Vuilleumier et al., 2007) or if it reduces sexual conflict (van Doorn & Kirkpatrick, 2007, 2010). Sex determination system turnover can even be caused by drift (Veller et al., 2017) or its interaction with sex ratio selection (Vuilleumier et al., 2007). Transitions between male and female heterogamety involving different sex determining chromosomes are widespread in many vertebrate groups (Ezaz et al., 2006; Pennell et al., 2018). However, switches of the heterogametic sex on the same chromosome (homologous transitions sensu van Doorn & Kirkpatrick, 2007) are less common, including only few known cases such as the frog *Rana rugosa* Temminck and Schlegel, 1838 (Miura, 2007; Ogata et al., 2008), *Neochromis* cichlids (Seehausen et al., 1999) and platyfish (Kallman, 1968).

In some species, male and female determiners on the same chromosome are found within a single population. Two different dominant female determiners occur on some X chromosomes in the Makobe Island population of the cichlid fish *Neochromis omnicaeruleus* Seehausen & Bouton, 1998 in Lake Victoria (Seehausen et al., 1999) and in multiple rodents (Veyrunes et al., 2010). Similarly, we find an effect of the recessive allele in both crosses with a sex determiner on chromosome P14/O9. In the Malawi cross, where we identified a dominant male determiner derived from *P. taeniolatus* (Y_TAE_), some individuals with a Y_TAE_ allele are female. In those XY females, the second allele is mostly derived from *A. calliptera* (X_CAL_) and only one carries an X_TAE_ allele (Figs. S3, S4). This may indicate that X_CAL_ has a feminizing effect. Similarly, in family B of the Victoria1 cross, we identified a dominant female determiner derived from *P*. sp. “nyererei-like” (W_NYL_). Most individuals with Z chromosomes derived from the same parental species (Z_PUL_Z_PUL_) are male (28/30). However, only seven individuals carry Z chromosomes derived from different parental species (Z_NYL_Z_PUL_) and only two of those are male (Figs. S3, S5). Therefore, Z_NYL_ could have a feminizing effect, but the notable paucity of Z_NYL_Z_PUL_ individuals in the Victoria1 family B (Fig. S3) more likely indicates reduced survival of males with two different Z chromosomes and thus the presence of incompatibilities.

Even though the Malawi and the Victoria1 crosses revealed the same sex chromosome with different heterogametic sexes, this case is likely not an example of homologous transition in heterogametic sex because the sex determiners do not represent direct sister states. At least four other chromosomes are sex determining in different cichlid species of Lake Malawi and, as our study highlights, other cichlid species from Lake Victoria also feature different sex determining chromosomes. It is thus likely that many switches between sex determining chromosomes occurred in the ancestry of the two species we here found to share the same sex chromosome. Our finding of different sex determiners on the same chromosome may rather support the hypothesis that certain chromosomes are more prone to carry sex determiners than other chromosomes as has been suggested for cichlids (Böhne et al., 2016), Ranidae frogs (Jeffries et al., 2018) and vertebrates in general (Marshall Graves & Peichel, 2010). These chromosomes may be enriched for genes contributing to the sex determination cascade or genes with sexually antagonistic effects (Blaser et al., 2014). Our study contributes to the accumulating information on loci involved in sex determination or sexual conflict in increasingly more species of the East African cichlid fish radiations, which will eventually allow us to test these hypotheses.

### Sex chromosome evolution

Male and female versions of sex chromosomes typically diverge over time as they accumulate neutral and deleterious mutations (Charlesworth, 1996; Bachtrog, 2013) and sexually antagonistic mutations (Charlesworth, 2017) on the non-recombining parts of the Y or W chromosome. However, we did not find a sex difference in sequencing depth in any cross, indicating that the sex chromosomes are not (yet) degenerated. The low divergence between the sex chromosomes (Fig. S6) indicates that they are very young, consistent with rapid turnover of sex chromosomes between species in these young radiations. The lack of sex chromosome degeneration may also facilitate further turnover of sex chromosomes as the accumulation of deleterious mutations is expected to stabilize sex determination systems (van Doorn & Kirkpatrick, 2010). This is because the invasion of a novel dominant sex determiner on another chromosome leads to a high proportion of individuals that are homozygous for the ancestral dominant sex determiner (YY or WW individuals) which have reduced fitness if deleterious mutations accumulated on this chromosome. Therefore, the lack of sex chromosome degeneration in the haplochromine cichlids may both be a consequence of rapid sex chromosome turnover and facilitate additional sex chromosome turnover.

## Conclusions

Overall, our results give further support to a growing body of evidence that sex determination in African cichlid fish is highly evolvable and may often involve several sex determiners with variable dominance relationships. Furthermore, switches between XY and ZW systems on the same chromosome seem to have occurred repeatedly. All of these factors may play a role in the adaptive radiations of East African cichlids. Studying additional species of the rapid radiations of haplochromine cichlids in Lakes Victoria, Malawi and also the smaller lakes in the region, will eventually allow us to better understand why there is such rapid turnover of sex determination systems in this species group and what the underlying mechanisms are. It will also allow us to find an answer to the question whether the rapid evolution of sex determination is more cause or more consequence of rapid species radiations. Cichlids are thus an ideal system to assess the importance of the evolution of sex determination in species diversification.

## Supplementary Information

Below is the link to the electronic supplementary material.

Supplementary material 1 (DOCX 2851 kb)

## Data Availability

Raw sequence data (fastq files) for all three crosses are available on the NCBI short read archive (SRA) under the following accession numbers: Victoria1 PRJNA612290 (from Feller et al. (2020a)); Victoria2 PRJNA439430 (from Feulner et al. (2018)); Malawi PRJNA612298 (from Feller et al. (2020b)). All files for QTL mapping and segregation pattern analyses and the code are available on the Dryad Digital Repository at 10.5061/dryad.b5mkkwhc8.
