# Supplementary material for: Identification of a novel sex determining chromosome in cichlid fishes that acts as XY or ZW in different lineages
Source: Hydrobiologia. 2021 Mar 13;848(16):3727–45. doi: 10.1007/s10750-021-04560-7 (PMC8550731; doi:10.1007/s10750-021-04560-7)
Supplement: Supplementary file 1 — Supplementary material 1 (DOCX 2851 kb) [file 10750_2021_4560_MOESM1_ESM.docx]

**Supplementary Figures and Tables**

**
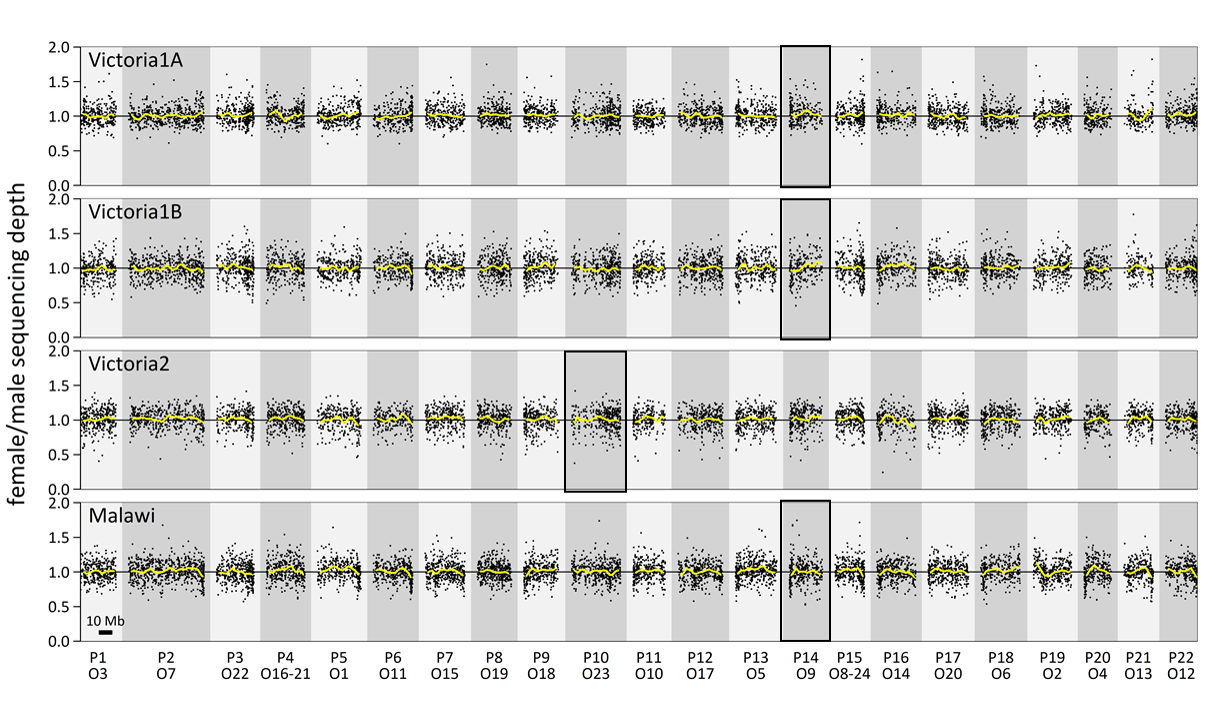
**

**Figure S1 | Sequencing depth ratios between sexes show no evidence for degenerated sex chromosomes.** Each dot shows the sequencing depth of females divided by the sequencing depth of males for a single SNP. The values are corrected for mean sequencing depth differences between the sexes to get a genome-wide average of 1. Yellow lines show 5 Mb window averages. Males and females show similar sequencing depths on all chromosomes, including the putative sex chromosomes (framed in black), which is inconsistent with strongly deteriorated Y or W chromosomes that would not map well to the reference genome.

**
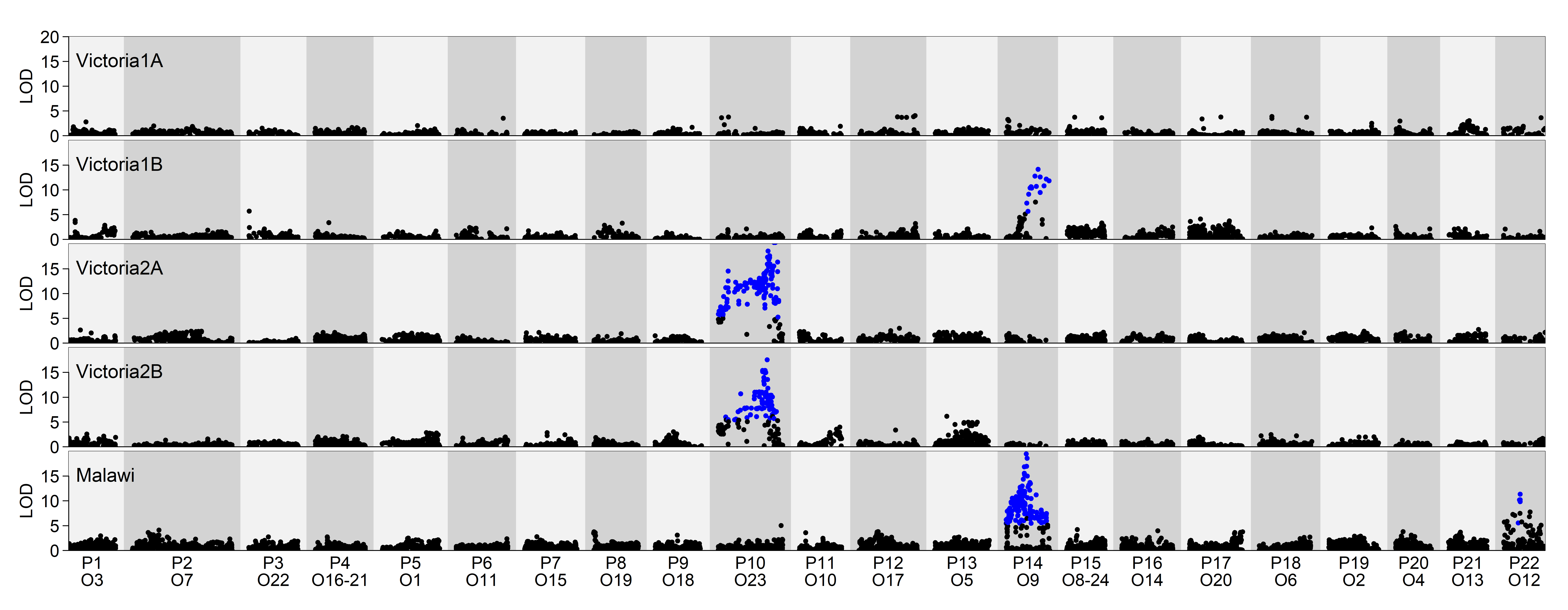
**

**Figure S2 | Single marker regressions**. Shown are LOD scores calculated from the F-statistic obtained in ANOVAs performed for each single marker (on x-axis). Highlighted in blue are scores associated with a p-value (from ANOVA) smaller than 0.05 after Bonferroni correction. The two families each of Victoria1 and Victoria2 are shown separately. In Victoria1, there is only a sex-association on chromosome P14/O9 in family A. In Victoria2, both families show similar patterns of a sex-association on chromosome P10/O23.

**
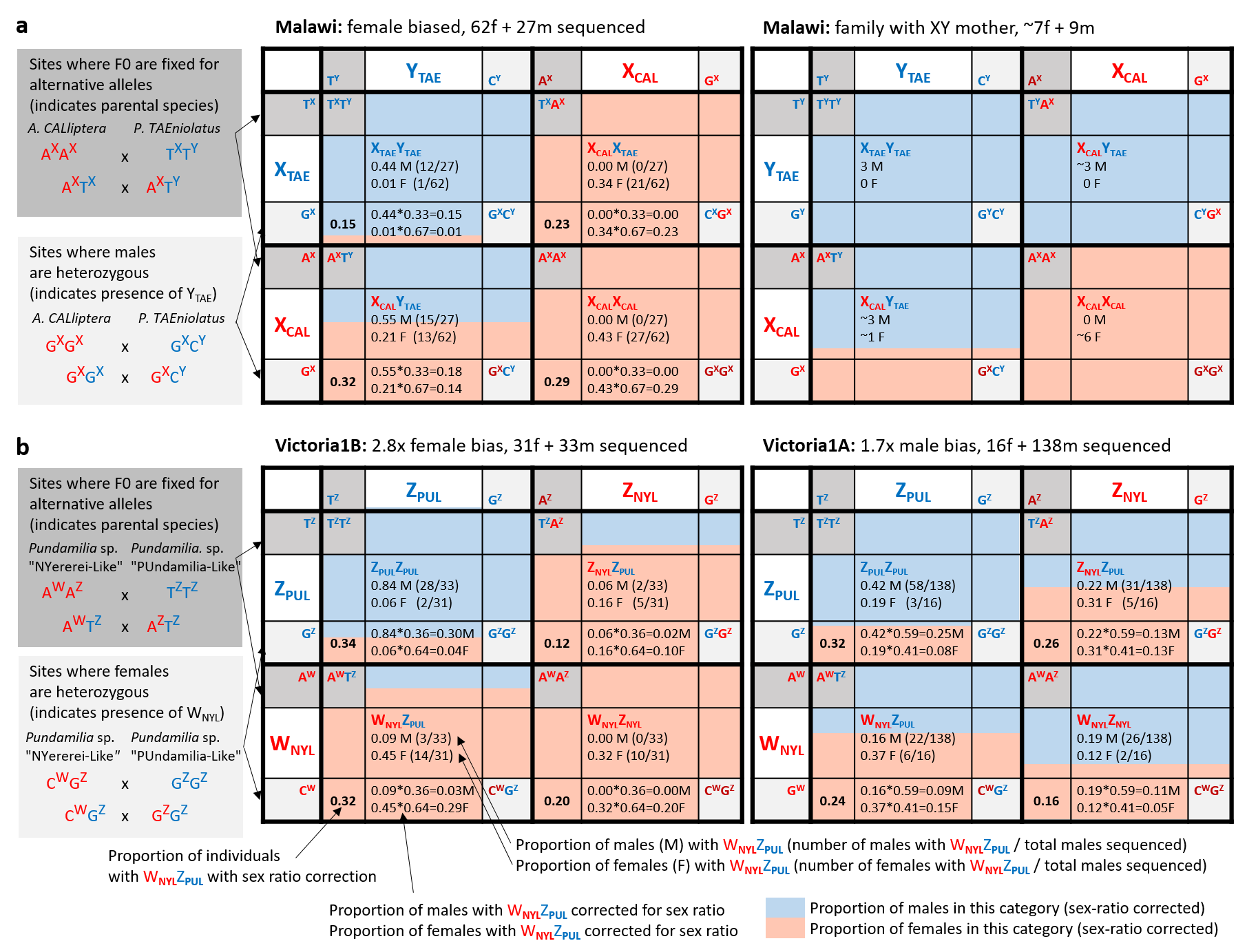
Figure S3 | Genotype proportions of males and females at the sex determining loci of the Malawi and the Victoria1 cross**

The genotype at the bi-allelic sites where the grandparents (F0) are fixed for alternative alleles indicate from which parental species the allele is derived. The genotypes at sites where the parents and grandparents of the heterogametic sex are heterozygous indicate the presence of the Y or W allele. These two categories are exemplified with an AT SNP for F0 fixed sites (dark grey) and for a CG SNP for sites heterozygous in the heterogametic sex. The combination of the genotypes at these sites, allows to infer the genotype of each individual at the sex-determining locus. Each of the four genotype categories in the Punnet squares should contain 25% of the individuals. However, given the biased sex ratios in the Malawi and the Victoria1 cross and given that we have sampled males and females at different proportions, we cannot simply add up the number of males and females in each category. Instead, we have computed the proportion of individuals in each genotype category (center of each category) for males and females separately. This proportion was then multiplied by the observed overall proportion of offspring of that sex (including unsequenced individuals) to get the expected genotype proportion if we had sampled the individuals randomly irrespective of sex (bottom of each category). The sum of these sex-ratio corrected proportions for males and for females together gives the corrected proportion of individuals in each category which should be 25%. The background shading roughly indicates the sex-ratio corrected proportion of males (blue) and females (red) in each category, *i.e.* the expected probability that an individual with this genotype will be male or female. **a** Genotype proportions at the most sex associated sites on chromosome P14/O9 (position 12,920,486 as site where F0 are fixed for alternative alleles and position 12,620,027 as site heterozygous in males). We do not know which individuals belong to which families, but three males showing a Y_TAE_Y_TAE_ genotype and 6 other recombinant males (see Fig. S4) indicate the presence of a family where both F1 parents had an X_CAL_Y_TAE_ genotype. Given that we have sequenced about twice as many females, we would expect about 6 X_CAL_X_CAL_ females from this family and one female with a X_CAL_Y_TAE_ genotype. This family is shown separately on the right. **b** Genotype proportions of Victoria1 family B (left) and family A (right). In family B the association of W_NYL_ with sex is much stronger than in family A. Notably, in family B, there are too few individuals with two different Z chromosomes (Z_NYL_Z_PUL_; top right category). Individuals in this category are expected to be male, but there are only two Z_NYL_Z_PUL_ males compared to 28 males with Z_PUL_Z_PUL_ (binomial test: p-value = 8.7e-7). See also Fig. S5.


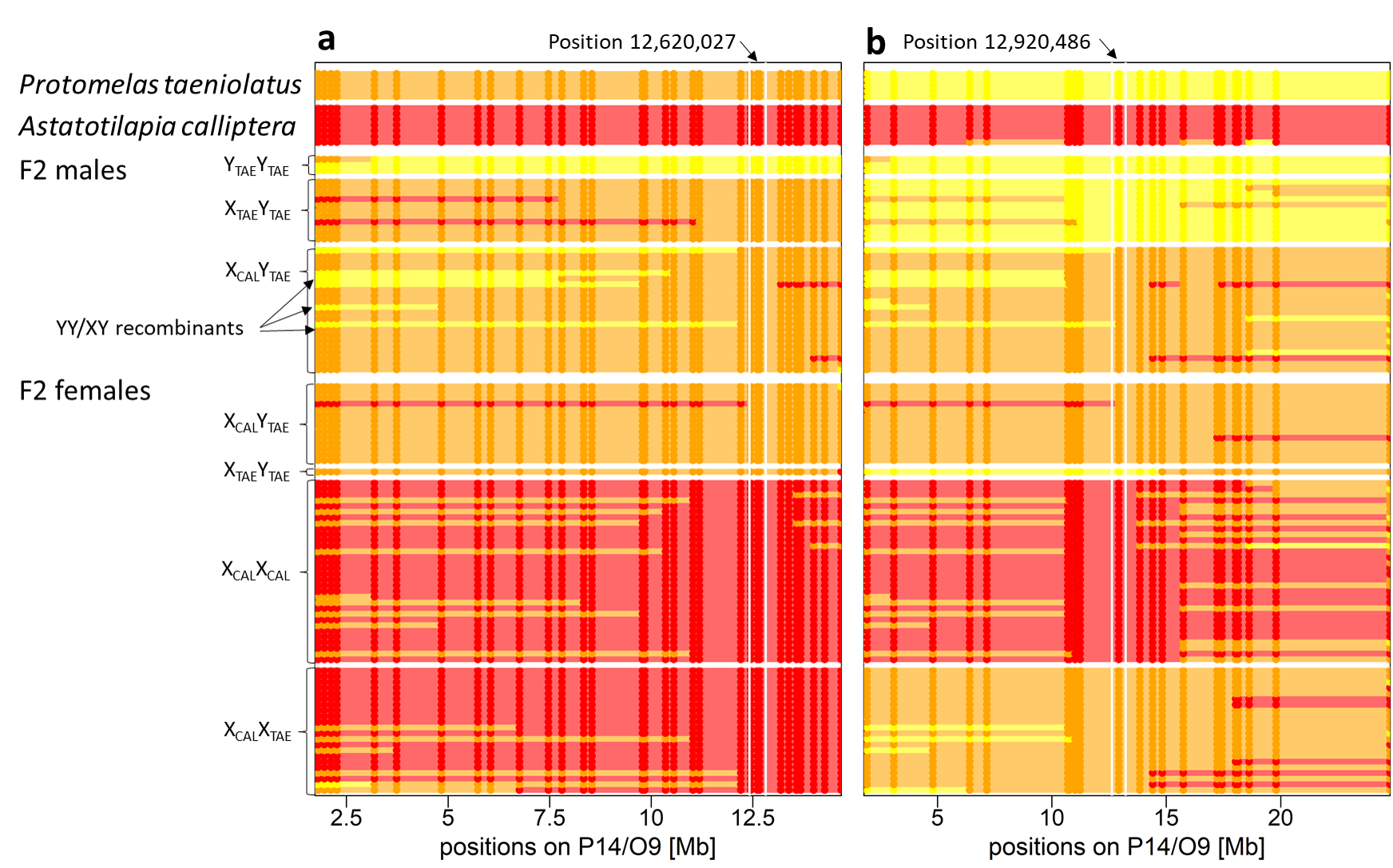


**Figure S4 | Visualization of the genotypes along the sex chromosome in the Malawi cross.**

**a** Sites where the grandparental surrogates, male *Protomelas taeniolatus*, are heterozygous and the grandmaternal surrogates, female *Astatotilapia calliptera*, are homozygous. These sites indicate the presence of a Y_TAE_ allele. Each individual is represented on a separate row with genotypes shown as red for XX, orange for XY and yellow for YY. The site used for the genotype proportions in Fig. S3 is indicated with a white frame. **b** Sites where the parental species have an allele frequency difference of at least 0.8. These sites allow to trace back the origin of the alleles to the parental species. The colours indicate parental species ancestry with homozygotes in yellow (TAE TAE) and red (CAL CAL) and heterozygotes (CAL TAE) in orange. The site used for Fig. S3 is framed in white.

**
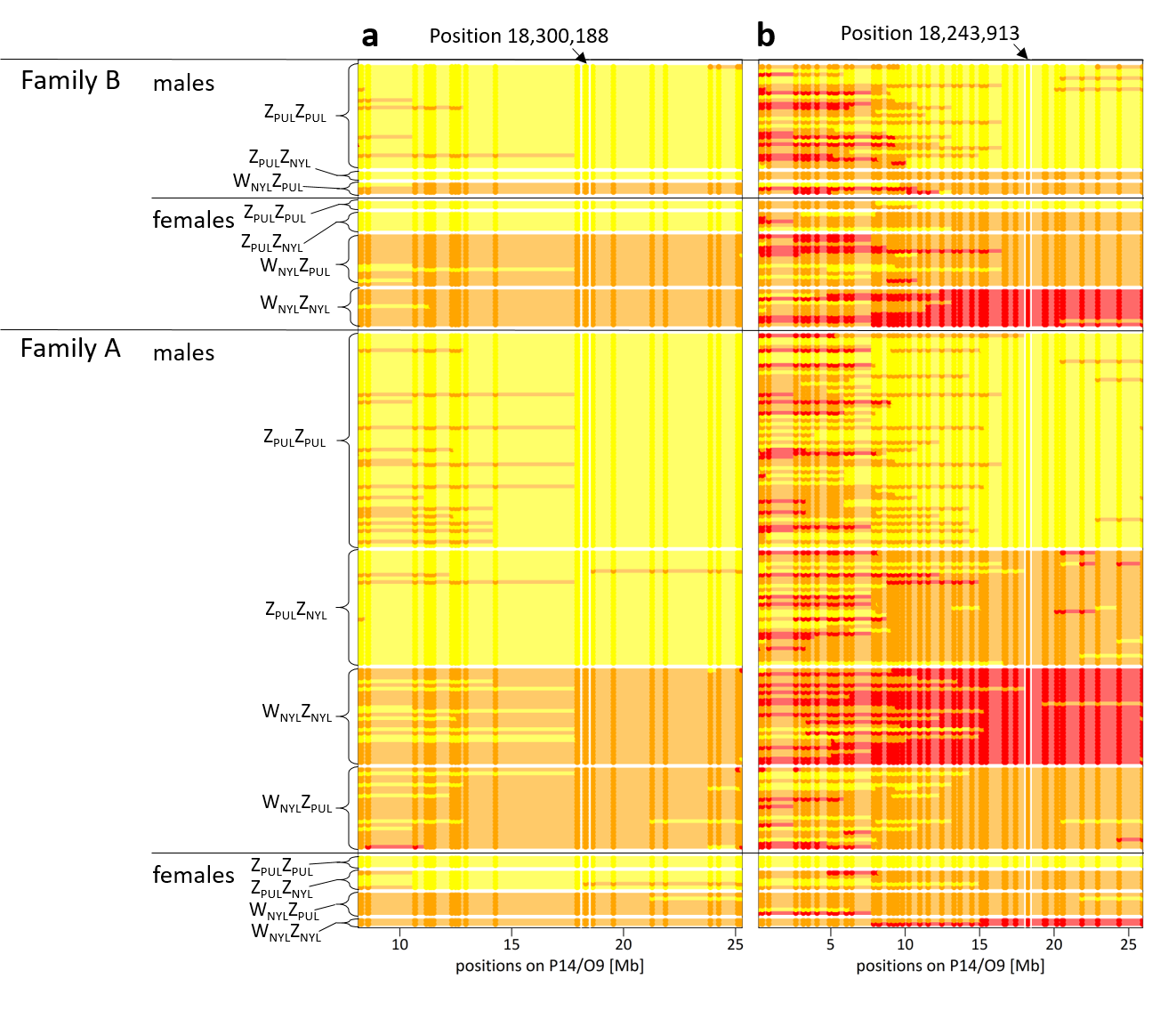
Figure S5 | Visualization of the genotypes along the sex chromosome in the Victoria1 cross.**

**a** Sites where grandmother and mothers are heterozygous and the grandfather and fathers are homozygous. These sites indicate the presence of a W_NYL_ allele. Each individual is represented on a separate row with genotypes shown as yellow for homozygotes of grandmaternal *P*. sp. “nyererei-like” allele (W_NYL_W_NYL_), orange for heterozygotes (W_NYL_Z) and yellow for ZZ. The site used for the genotype proportions in Fig. S3 is indicated with a white frame. **b** Sites where the grandparents are homozygous for alternative alleles and the parents are heterozygous. These sites allow to trace back the origin of the alleles to the parental species. The colours indicate parental species ancestry with homozygotes in yellow (NYL NYL) and red (PUL PUL) and heterozygotes (NYL PUL) in orange. The site used for Fig. S3 is framed in white.


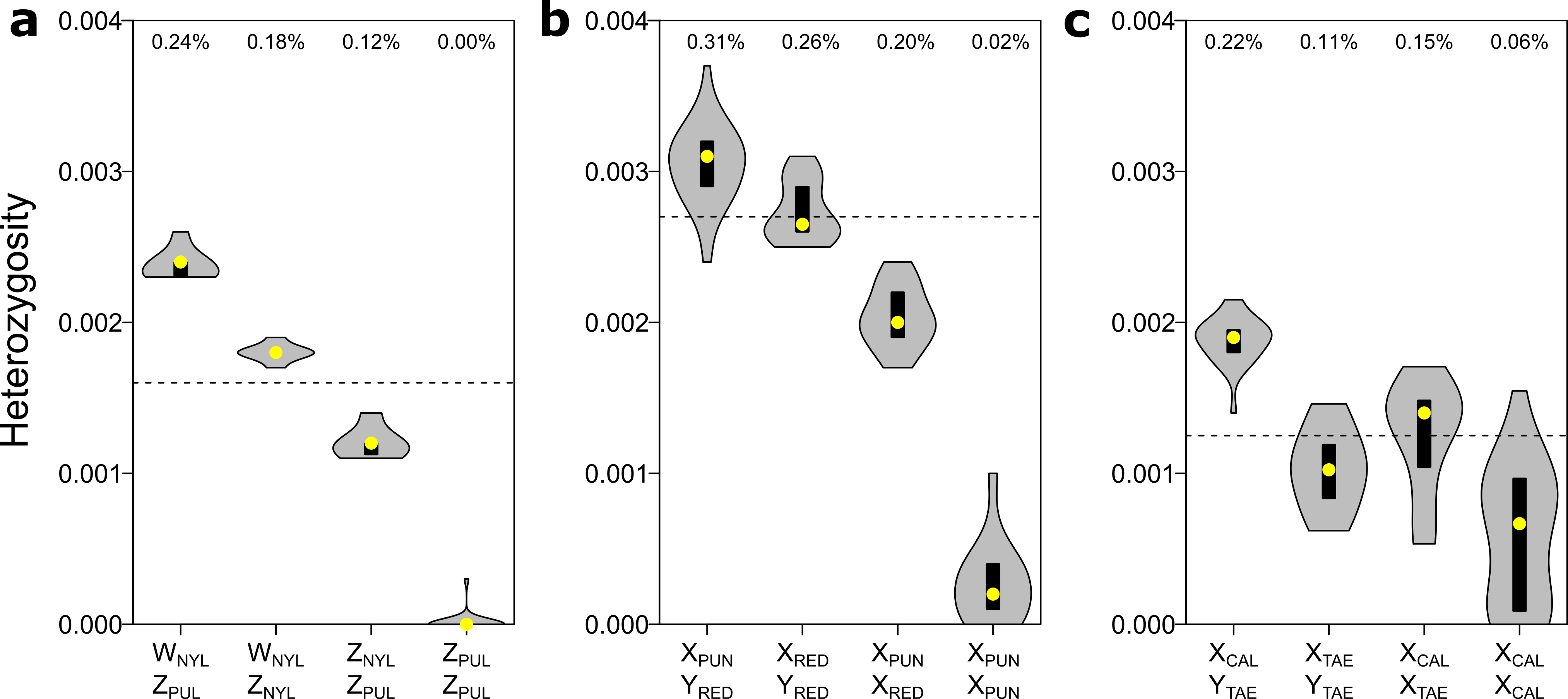


**Figure S6 | Low divergence between sex chromosomes in all three crosses**

Divergence between sex chromosomes was computed as heterozygosity (number of heterozygous sites among all sites sequenced) at the chromosomal region with strongest sex association. Density kernels (violin plots) show the heterozygosity distributions of all individuals with a specific combination of sex chromosomes indicated below. The median given on top is indicated as a yellow dot and the interquartile range is shown as a black box. The dashed horizontal line corresponds to the divergence (d_xy_) between Victoria 1 and Victoria 2 individuals and thus represents a divergence time of maximum 15,000 years. **A)** Heterozygosity at the P14/O9 chromosome at 18-19 Mb in the Victoria1 cross, family B. The WZ chromosomes are derived from the *P.* sp. “nyererei-like” (NYL) grandmother or the *P*. sp. “pundamilia-like” (PUL) grandfather. The low heterozygosity in Z_PUL_Z_PUL_ individuals suggests that the mother and father both inherited the same Z chromosome from the grandfather. **B)** Heterozygosity at the P10/O23 chromosome at 30-31 Mb in the Victoria2 cross. The chromosomes are derived from the *P.* *pundamilia* (PUN) grandmother or the *P*. sp. “red-head” grandfather (RED). **C)** Heterozygosity at the P14/O9 chromosome at 11-14 Mb in the Malawi cross, with chromosomes derived from the *A. calliptera* (CAL) grandmother or the *P. taeniolatus* (TAE) grandfather. Note that in all three crosses, the heterozygosity is higher in heterogametic (XY/ZW) than in homogametic (XX/ZZ) individuals, and highest in heterogametic individuals with sex chromosomes from different parental species. However, the divergence between different sex chromosomes is not much greater than the divergence between the individuals of the two Lake Victoria cichlid crosses which have diverged maximum 15,000 years ago.

| **Table S1.** QTL mapping results (only results with p-values <0.1 shown) | | | | | | | | | | | |
| --- | --- | --- | --- | --- | --- | --- | --- | --- | --- | --- | --- |
|  | #F2 m/f | marker  (nearest) | P-LG | pos (cM) | 95% CI | nearest flanking markers | O-LG | LOD | p-value | PVE |  |
| Victoria2 | 130/56 | c10.loc18  chr10_28361037 | 10 | 18.00 | 16.00-25.00 | chr10_27536410-chr10_28361037 | 23 | 21.58 | 0.00 | 41.39 |  |
| Victoria2; add | 121/56 | chr10_28361037 | 10 | 17.90 |  |  | 23 | 20.20 | 0.00 |  |  |
| Victoria2; famA | 76/47 | c10.loc19 (chr10_28361037) | 10 | 19.00 |  |  | 23 | 13.80 | 0.00 |  |  |
| Victoria2; famB | 45/9 | chr10_26890500 | 10 | 15.20 |  |  | 23 | 6.64 | 0.00 |  |  |
| Malawi | 36/69 | chr14_11239278 | 14 | 16.97 | 13.00-20.27 | chr14_7158496-chr14_12920486 | 9 | 11.96 | 0.00 | 40.84 |  |
| #F2 m/f, number of F2 males/females used in QTL mapping; (nearest), nearest RAD marker to a QTL at an interpolated marker (where genotypes were inferred with the calc.genoprob function in R/qtl); P-LG, linkage group number corresponding to the anchored *Pundamilia nyerere*i reference; O-LG, linkage group number corresponding to the *Oreochromis niloticus* reference; pos (cM), position in centiMorgan; 95% CI, 95% approximate Bayesian credible interval (in cM); PVE, percent variance explained; add, accounting for family as additive covariate; famA and famB, separate analyses for the two families. | | | | | | | | | | | |
